# CD4^+^CCR6^+^ T cells, but not γδ T cells, are important for the IL‐23R‐dependent progression of antigen‐induced inflammatory arthritis in mice

**DOI:** 10.1002/eji.201948112

**Published:** 2019-11-28

**Authors:** Wida Razawy, Patrick S. Asmawidjaja, Anne‐Marie Mus, Nazike Salioska, Nadine Davelaar, Nicole Kops, Mohamed Oukka, C. Henrique Alves, Erik Lubberts

**Affiliations:** ^1^ Department of Rheumatology Erasmus MC University Medical Center Rotterdam The Netherlands; ^2^ Department of Immunology University Medical Center Rotterdam The Netherlands; ^3^ Department of Orthopaedics, Erasmus MC University Medical Center Rotterdam The Netherlands; ^4^ Department of Pediatrics Seattle Children's Research Institute Center for Immunity and Immunotherapies Seattle USA; ^5^ Department of Immunology University of Washington Seattle USA

**Keywords:** IL‐23, IL‐17, CCR7, T cells, arthritis

## Abstract

IL‐23 plays an important role in the development of arthritis and the IL‐23 receptor (IL‐23R) is expressed on different types of T cells. However, it is not fully clear which IL‐23R^+^ T cells are critical in driving T cell‐mediated synovitis. We demonstrate, using knock‐in IL‐23R‐GFP reporter (IL‐23R^GFP/+^) mice, that CD4^+^CCR6^+^ T cells and γδ T cells, but not CD8^+^ T cells, express the IL‐23R(GFP). During early arthritis, IL‐23R(GFP)^+^CD4^+^CCR6^+^ T cells, but not IL‐23R(GFP)^+^ γδ T cells, were present in the inflamed joints. IL‐23R^GFP/+^ mice were bred as homozygotes to obtain IL‐23R^GFP/GFP^ (IL‐23R deficient/IL‐23R^−/−^) mice, which express GFP under the IL‐23R promotor. Arthritis progression and joint damage were significantly milder in IL‐23R^−/−^ mice, which revealed less IL‐17A^+^ cells in their lymphoid tissues. Surprisingly, IL‐23R^−/−^ mice had increased numbers of IL‐23R(GFP)^+^CD4^+^CCR6^+^ and CCR7^+^CD4^+^CCR6^+^ T cells in their spleen compared to WT, and IL‐23 suppressed CCR7 expression in vitro. However, IL‐23R(GFP)^+^CD4^+^CCR6^+^ T cells were present in the synovium of IL‐23R^−/−^ mice at day 4. Finally, adoptive transfer experiments revealed that CD4^+^CCR6^+^ T cells and not γδ T cells drive arthritis progression. These data suggest that IL‐23R‐dependent T cell‐mediated synovitis is dependent on CD4^+^CCR6^+^ T cells and not on γδ T cells.

## Graphical abstract

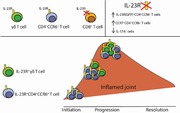

## Introduction

The IL‐23 signaling pathway has been implicated in different autoimmune diseases including rheumatoid arthritis, multiple sclerosis, inflammatory bowel disease, and psoriasis 1, 2. IL‐23 is mainly produced by myeloid cells such as dendritic cells (DCs) and macrophages 3, and belongs to the IL‐12 cytokine family. Similarly to the other family members, both the cytokine and its receptor act as heterodimers. In this context, functional IL‐23 consists of a complex formed by IL‐23p19, which binds the IL‐23R subunit, and IL‐12p40, which binds the IL‐12Rβ1 subunit of the IL‐23 receptor 4, 5, 6. IL‐23R signals through the STAT3 pathway resulting in pro‐inflammatory cytokine production including IL‐17A, IL‐22, IFNγ, GM‐CSF, the expression of the chemokine receptor CCR6 and the transcription factor RORγt 7. While RORγt is crucial for driving the differentiation of IL‐17‐producing T cells 8, regulation of CCR6 expression is important for their migration towards the site of inflammation 9, 10.

The IL‐23R is expressed mainly on γδ T cells and on a small fraction of CD4^+^ T cells, DCs and macrophages in the LNs of mice during steady state 11. However, the dynamics of IL‐23R^+^ T cells is different during inflammatory conditions. During experimental autoimmune encephalomyelitis (EAE), IL‐23R^+^ CD4^+^ T cells were increased compared to naïve condition and accumulated in the inflamed CNS of mice 11. Similarly, IL‐23R^+^ γδ T17 cells accumulated in the skin‐draining LNs of mice during Aldara‐induced skin inflammation 12. However, the kinetics of different IL‐23R^+^ T cells during arthritic versus steady state conditions is not fully investigated.

Previously, it was shown that mice deficient in IL‐23p19 were resistant to collagen‐induced arthritis 13, 14. We demonstrated that the progression of antigen‐induced arthritis (AIA) is dependent on IL‐23 and IL‐17RA signaling, since both IL‐23p19^−/−^ and IL‐17RA^−/−^ mice had significantly milder arthritis compared to wild type (WT) 15. While, in the joints of arthritic mice, both IL‐17A producing CD4^+^ T cells and γδ T cells were detected, there was a significant decrease in these cells in the absence of IL‐23. This suggests that both cell types play a role in IL‐23‐mediated joint inflammation. It should be noted that discrepancy exists between IL‐17A KO and IL‐17RA^−/−^ mice regarding the severity of AIA, since IL‐17A KO mice showed no difference in AIA severity 16, suggesting that other IL‐17 family members are involved.

Nevertheless, although the role of Th17 cells in IL‐23‐dependent arthritis has been extensively studied and well‐appreciated 14, 17, 18, the relative significance of IL‐23R^+^ γδ T cells remains elusive 17, 19, 20, 21, 22, 23.

In the present study, we identified different types of IL‐23R^+^ T cells in the joint draining LNs and the joints during the course of AIA, using IL‐23R‐GFP reporter mice. Furthermore, we induced AIA in WT and IL‐23R^−/−^ mice and analyzed T cells in their joints and spleens during the peak of disease. Lastly, we investigated which type of T cells are crucial for driving IL‐23R‐dependent joint inflammation.

## Results

### IL‐23R(GFP)^+^CD4^+^ T cells, but not γδ T cells, are present in the joints during early arthritis

**Figure 1:**
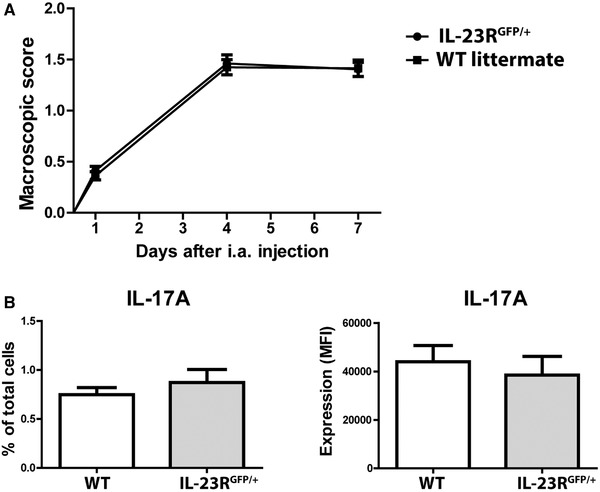
IL‐23R‐GFP reporter and WT mice have similar susceptibility to AIA. AIA was induced in IL‐23R^GFP/+^ and WT mice, and mice were sacrificed at days 1, 4, or 7 after arthritis induction. (A) Macroscopic scores of joint inflammation. Pooled data of two independent experiments are depicted for day 1 (*n* = 5 mice per group), day 4, and day 7 (*n* = 8 mice per group). (B) IL‐17A production assessed by flow cytometry in the spleen at day 4 of AIA after stimulation of cells for 4 h with PMA/ionomycin. MFI = mean fluorescent intensity. Representative data of two independent experiments given for *n* = 4 mice per group per experiment. Data are depicted as mean with SEM and compared using Mann–Whitney test. *p*‐values <0.05 were considered statistically significant.

We aimed to identify IL‐23R expressing cells using IL‐23R reporter (IL‐23R^GFP/+^) mice in which the intracellular domain of the IL‐23R is replaced by GFP 11. Since IL‐23R^GFP/+^ mice have only one functional *il23r* allele, we first assessed the severity of arthritis in these mice by macroscopically assessing joint inflammation at day 1, 4 or 7 after the induction of arthritis. Interestingly, both the onset and the progression of arthritis in IL‐23R^GFP/+^ mice were similar to WT controls (Fig. 1A). Furthermore, the lymphoid cells of both groups were equally capable of producing the pro‐inflammatory cytokines IL‐17A and IL‐17F (Fig. 1B and Supporting Information Fig. 1).

**Figure 2:**
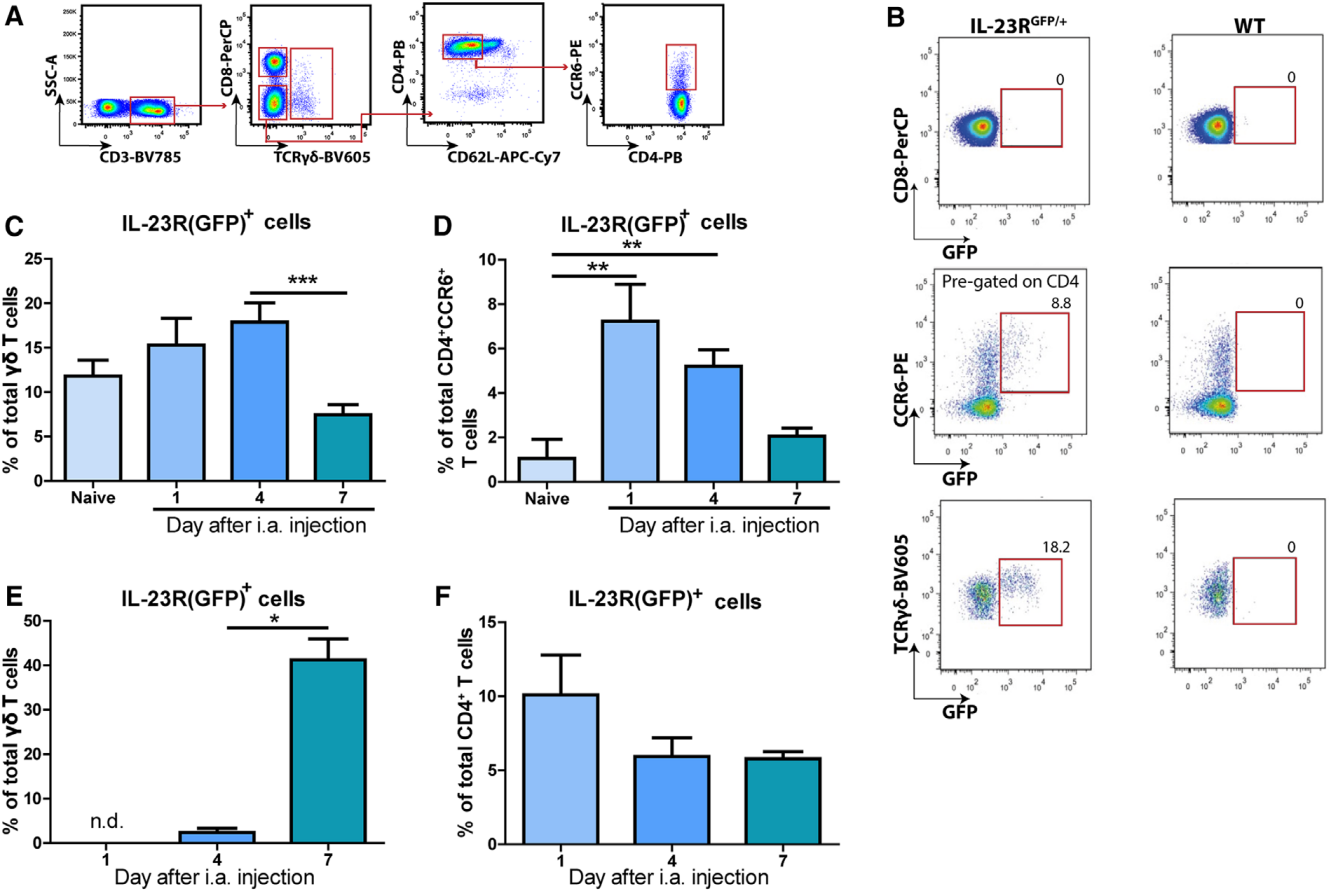
IL‐23R(GFP)^+^CD4^+^ T cells are present in the pLNs and joints during the early stages of arthritis. AIA was induced in IL‐23R^GFP/+^ and WT mice. Naïve and arthritic mice were sacrificed at days 1, 4, or 7 of AIA. IL‐23R(GFP) expression was assessed by flow cytometry. WT mice were used as negative control for GFP signal. (A) Representative gating strategy for the analysis of T cells. Cells were pre‐gated as indicated in Supporting Information Fig. 2. (B) Representative plot of IL‐23R(GFP)expression at day 1 of AIA in the LNs. CCR6^+^ cells were pre‐gated for CD4 as depicted in (A). (C‐D) Quantification of (B) for IL‐23R(GFP)^+^ (C) γδ T cells or (D) CD4^+^CCR6^+^ T cells in the pLNs of IL‐23R^GFP/+^ mice. (E‐F) IL‐23R(GFP)^+^ (E) γδ T cells or (F) CD4^+^ T cells in the joints of IL‐23R^GFP/+^ mice. (E‐F) Cells were pre‐gated on CD45 and CD3. Pooled data of two independent experiments for naïve mice (*n* = 7 mice per group), AIA day 1 (*n* = 5 mice per group), and three independent experiments for AIA day 4 (*n* = 10 mice per group) and day 7 (*n* = 12 mice per group) are depicted as mean with SEM. *p < 0.05, **p < 0.01, ***p < 0.001 (*Kruskall–Wallis test*).

Since the synovial inflammation in the AIA model is dependent on T cells, we aimed to identify different types of IL‐23R^+^ T cells in this model. Therefore, we analyzed IL‐23R(GFP) expression in the T cells of the popliteal LNs (pLNs) of mice, which drain from the joints (Fig. 2A and Supporting Information Fig. 2). IL‐23R(GFP) expression was found in γδ T cells and CD4^+^ T cells during both naïve and arthritic conditions, but not in CD8^+^ T cells (Fig. 2B). Activated T cells downregulate the expression of the adhesion molecule CD62L 24. Notably, IL‐23R(GFP) expression within the CD4^+^CD62L^−^ (effector/effector memory) T cell population was confined to only CCR6^+^ cells, referred to as CD4^+^CCR6^+^ T cells in this paper.

Next, we assessed IL‐23R(GFP) expression in the T cells of the pLNs and the joints during the different phases of arthritis. In the pLNs of naïve mice, approximately 12% of γδ T cells expressed the IL‐23R(GFP) (Fig. 2C). The fraction of these cells increased slightly at days 1 and 4, but decreased significantly at day 7. In contrast to the pLNs, IL‐23R(GFP)^+^ γδ T cells could not be detected in the joints at day 1 of AIA (Fig. 2E). However, a small proportion of γδ T cells was IL‐23R(GFP)^+^ at day 4 of AIA and increased significantly by day 7.

In naïve pLNs, around 1% of CD4^+^CCR6^+^ T cells expressed the IL‐23R(GFP) (Fig. 2D). However, the fraction of these cells increased significantly already at day 1 of arthritis, but decreased gradually during the peak of disease in the pLNs. Also, in the spleen and inguinal LNs, which drain from the site of immunization, the percentage of IL‐23R(GFP) expressing CD4^+^CCR6^+^ T cells was increased during arthritis compared to naïve condition (data not shown). In contrast to γδ T cells, IL‐23R(GFP)^+^CD4^+^CCR6^+^ T cells could be detected in the joints of mice at day 1 of AIA (Fig. 2F), but their fraction slightly decreased during days 4 and 7. These data demonstrate that the kinetics of IL‐23R(GFP)^+^ γδ T cells differ from that of CD4^+^CCR6^+^ T cells in the pLNs and the joints during the course of arthritis. Furthermore, IL‐23R(GFP)^+^CD4^+^CCR6^+^ T cells are present at the site of inflammation during the early phases of arthritis.

### The progression of full‐blown inflammatory arthritis is dependent on IL‐23R signaling

**Figure 3:**
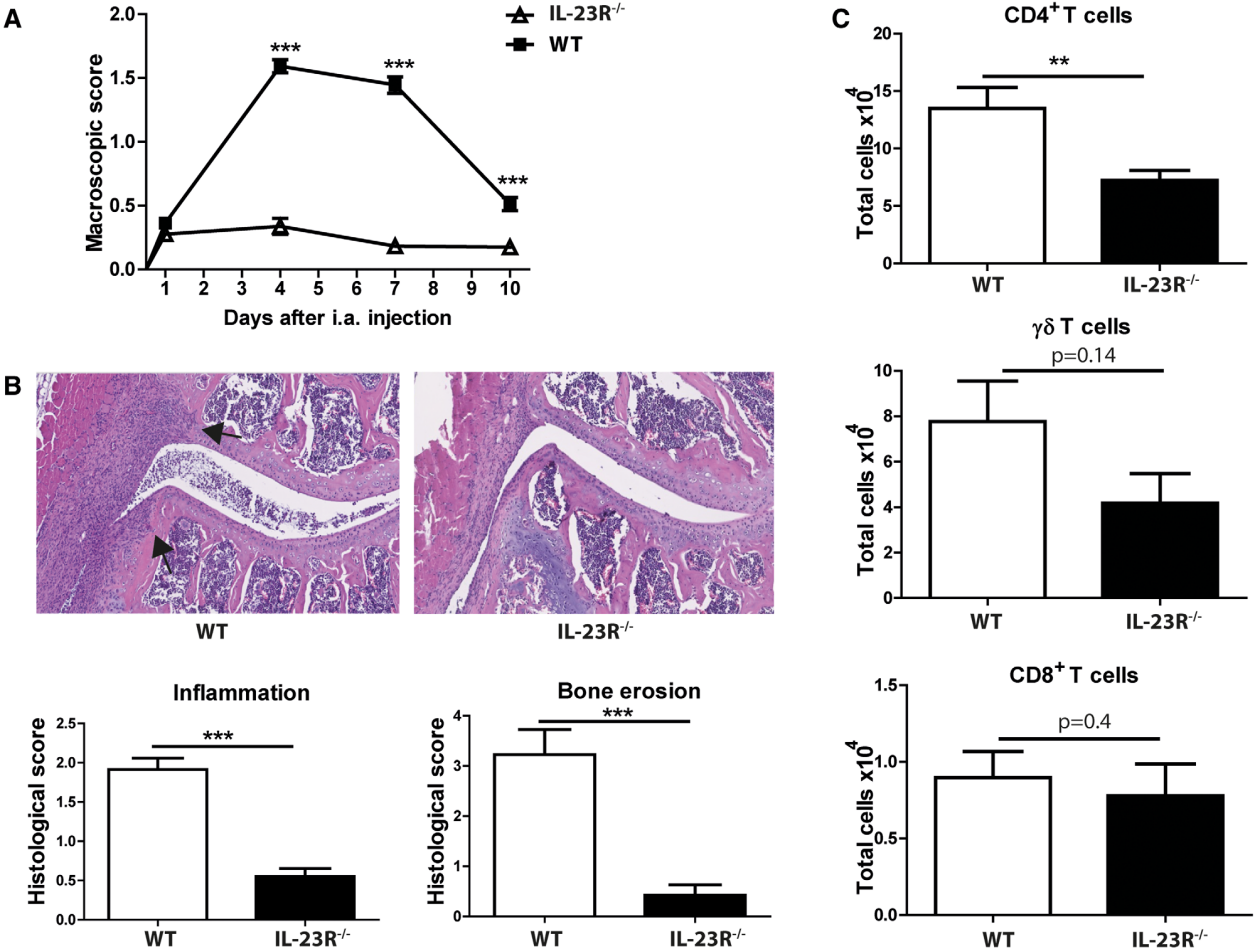
IL‐23R signaling is important during the progressive phase of arthritis. AIA was induced in WT and IL‐23R^−/−^ mice, and mice were sacrificed at days 1, 4, 7, or 10 after arthritis induction. (A) Macroscopic scores of joint inflammation. (B) Representative hematoxylin and eosin (H&E) staining and histologic scores of the knee joints at day 10 of AIA. Arrows indicate bone damage, scale 1:300. (C) Total CD4^+^, TCRγδ^+^, and CD8^+^ T cell counts in the joints of mice at day 4 of AIA assessed by flow cytometry. Cells were pre‐gated on CD45 and CD3. Pooled data of two independent experiments with *n* = 5 mice per group for AIA day 1 and day 10, and three independent experiments for AIA day 4 (*n* = 10 mice per group) and day 7 (*n* = 12 mice per group) are depicted as mean with SEM for per group. **p < 0.01, ***p < 0.001 (*Mann–Whitney test*).

When IL‐23R^GFP/+^ mice are bred as homozygotes, IL‐23R^GFP/GFP^ mice can be obtained which are deficient for IL‐23R signaling (IL‐23R^−/−^ mice). To study the role of IL‐23R signaling in a T cell‐mediated inflammatory arthritis, we induced AIA in IL‐23R^−/−^ and WT mice, and monitored joint inflammation via macroscopic scoring at days 1, 4, 7 and 10 after arthritis induction. Although the onset of AIA was similar in both groups, the progression of joint inflammation was significantly lower in IL‐23R deficient mice (Fig. 3A). In line with the macroscopic scores, histological analysis of the knee joints revealed significantly less joint inflammation and bone damage in IL‐23R^−/−^ mice at day 10 (Fig. 3B).

Since IL‐23R^−/−^ joints were less inflamed compared to WT, we investigated if there were differences in T cell infiltration. Therefore, we analyzed T cells in the joints by flow cytometry at day 4 of AIA. Interestingly, total CD4^+^ T cell numbers were significantly reduced in IL‐23R^−/−^ joints (Fig. 3C). Furthermore, γδ T cell counts were lower compared to WT, although this was not statistically significant. In contrast, CD8^+^ T cell numbers were comparable between both groups, and total CD8^+^ T cell numbers were low compared to CD4^+^ T cells and γδ T cells. Altogether, these data indicate that IL‐23R signaling is crucial for the progressive phase, but not for the onset of AIA and suggest that T cell infiltration in the inflamed joints plays an important role during the progression of arthritis.

### IL‐23R(GFP)^+^CD4^+^CCR6^+^ T cells are increased in the lymphoid tissues of IL‐23R deficient mice

**Figure 4:**
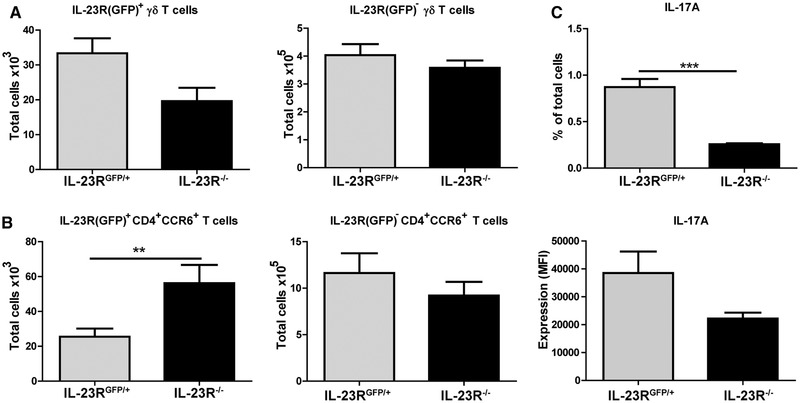
Total IL‐23R(GFP)^+^CD4^+^CCR6^+^ T cell numbers are higher in IL‐23R deficient mice compared to WT. AIA was induced in WT, IL‐23R^GFP/+^, and IL‐23R^−/−^ mice and the spleens were harvested at day 4 after arthritis induction. Total IL‐23R^+^ T cell count was determined using GFP expression by flow cytometry. WT mice were used as negative control for GFP. (A) Total IL‐23R(GFP)^+^ and IL‐23R(GFP)^−^ γδ T cell and (B) CD4^+^CCR6^+^ T cell numbers. Pooled data of three independent experiments are depicted for *n* = 10–12 mice per group. (C) % IL‐17A^+^ cells and IL‐17A MFI in all cells assessed by flow cytometry in the spleen at day 4 of AIA. MFI = mean fluorescent intensity. Representative data of two independent experiments given for *n* = 4 mice per group per experiment. Data are depicted as mean with SEM. **p < 0.01, ***p < 0.001 (*Mann–Whitney test*).

The previous data suggested that CD4^+^ T cell infiltration in the joints is abrogated in the absence of IL‐23R signaling. To investigate if this is caused by any alterations in T cells in the lymphoid tissues, we analyzed both IL‐23R(GFP)^+^ and IL‐23R(GFP)^−^ T cells in the spleens of the mice at day 4 of AIA. Total cell numbers of IL‐23R(GFP) expressing γδ T cells were slightly lower in the spleens of IL‐23R^−/−^ mice compared to IL‐23R^GFP/+^ mice (Fig. 4A). In contrast, IL‐23R(GFP)^+^CD4^+^CCR6^+^ T cells were significantly higher in the spleens (Fig. 4B) and inguinal LNs (Supporting Information Fig. 3) of IL‐23R^−/−^ mice compared to IL‐23R^GFP/+^. Total cell count of IL‐23R(GFP)^−^ γδ T cells and IL‐23R(GFP)^−^CD4^+^CCR6^+^ T cells were similar between both groups (Fig. 4A and B and Supporting Information Fig. 3). Despite the increase in IL‐23R(GFP)^+^CD4^+^CCR6^+^ T cells, splenic cells of IL‐23R^−/−^ mice were less capable of producing IL‐17A and IL‐17F (Fig. 4C and Supporting Information Fig. 4). These findings suggest that IL‐23R(GFP)^+^CD4^+^CCR6^+^ T cells have decreased inflammatory potential and accumulate in the lymphoid tissues of IL‐23R^−/−^ mice during arthritis.

A potential explanation for the accumulation of the IL‐23R(GFP)^+^CD4^+^CCR6^+^ T cells in the lymphoid tissues of IL‐23R deficient mice could be abnormalities in their migratory capacity. Effector T cells migrate from lymphoid tissues towards the site of inflammation under the influence of chemokines and chemokine receptors. To investigate if chemokine receptor expression is altered in the absence of IL‐23R signaling, we analyzed CCR6 and CCR7 expression in the spleens of the mice at day 4 of AIA. The expression of CCR6, which is associated with T cell migration towards the joints 9, was not different between IL‐23R(GFP)^+^CD4^+^CCR6^+^ T cells in both groups (Supporting Information Fig. 5A). Thus, this does not explain the increase in the number of these cells in the spleens.

**Figure 5:**
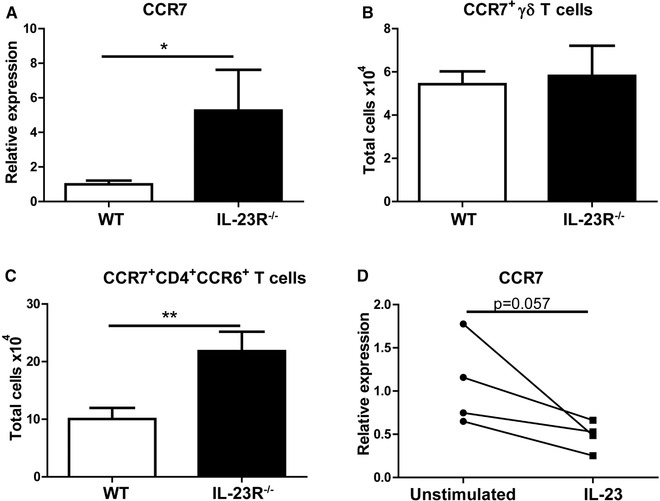
CCR7^+^CD4^+^CCR6^+^ T cells are increased in the spleens of arthritic IL‐23R^−/−^ mice. AIA was induced in WT and IL‐23R^−/−^ mice and the spleens were harvested at day 4 after arthritis induction. (A) CCR7 gene expression in the splenocytes was assessed by RT‐PCR. (B) Total cell numbers of CCR7^+^ γδ T cell or (C) CD4^+^CCR6^+^ T cells were assessed by flow cytometry. Cells were pre‐gated as depicted as shown in Fig. 2A. (A–C) Pooled data of two independent experiments with *n* = 7–10 mice per group. (D) Splenic cells of WT mice were cultured for 3 days with or without IL‐23 and CCR7 gene expression was assessed by RT‐PCR. Data with *n* = 4 mice per group. Data are depicted as mean with SEM. *p < 0.05, **p < 0.01 (*Mann–Whitney test*).

Next, we focused on CCR7, which is downregulated on activated T cells during their egress from lymphoid tissues towards the site of inflammation 25. Analysis of CCR7 gene expression revealed almost six fold higher expression in IL‐23R^−/−^ splenocytes compared to WT (Fig. 5A). In order to further confirm this, we analyzed CCR7 protein levels by flow cytometry. Total number of CCR7 expressing γδ T cells was similar between WT and IL‐23R deficient mice (Fig. 5B). In contrast, CCR7^+^CD4^+^CCR6^+^ T cells were significantly higher in IL‐23R^−/−^ spleens (Fig. 5C). However, CCR7 MFI on CD4^+^CCR6^+^ T cells was not significantly different between both groups (Supporting Information Fig. 5B). To confirm that IL‐23 has a role in the regulation of CCR7 expression, we stimulated splenocytes of WT mice for 3 days with IL‐23. CCR7 expression was reduced in the splenocytes of all mice upon treatment with IL‐23 (Fig. 5D).

Overall, these data suggest that IL‐23R signaling plays a role in the regulation of CCR7 expression on CD4^+^CCR6^+^ T cells.

### IL‐23R(GFP)^+^ γδ T cells and CD4^+^CCR6^+^ T cells are present in the joints of arthritic IL‐23R^−/−^ mice

**Figure 6:**
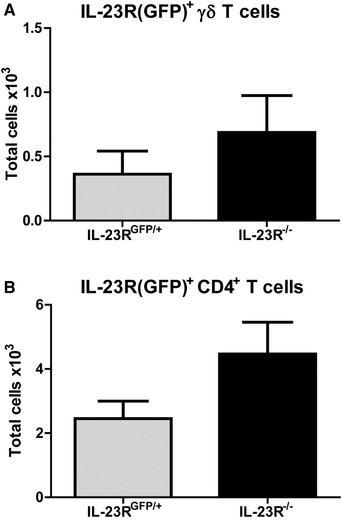
IL‐23R(GFP)^+^CD4^+^ T cells and γδ T cells are present in the joints of arthritic IL‐23R^−/−^ mice. AIA was induced in WT, IL‐23R^GFP/+^, and IL‐23R^−/−^ mice and the joints were harvested at day 4 of AIA. Total IL‐23R^+^ T cell count was determined using GFP expression by flow cytometry. WT mice were used as negative control for GFP. (A) Total IL‐23R(GFP)^+^ γδ T cell and (B) CD4^+^ T cell numbers. Data are representative of three independent experiments and depicted as mean with SEM for *n* = 4 mice per group per experiment and compared using Mann–Whitney test. *p*‐values < 0.05 were considered statistically significant.

The previous data suggested that IL‐23R(GFP)^+^CD4^+^CCR6^+^ T cells accumulated in the lymphoid tissues of IL‐23R deficient mice at the peak of disease (Fig. 4 and 5). Therefore we set out to analyze these cells in the joints of mice at this time point. Interestingly, both IL‐23R(GFP) expressing γδ T cells and CD4^+^CCR6^+^ T cells were present in the joints of IL‐23R deficient mice and were even slightly higher than in IL‐23R^GFP/+^ joints (Fig 6A and B). These data suggest that IL‐23R^+^ T cells are able to enter the site of inflammation even in the absence of IL‐23R signaling.

### CD4^+^CCR6^+^ T cells, but not γδ T cells, are important for IL‐23R‐dependent progression of arthritis

**Figure 7:**
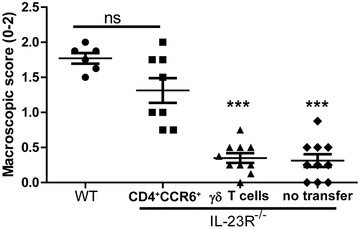
CD4^+^CCR6^+^ T cells, but not γδ T cells, are important for the progressive phase of AIA. WT mice were immunized with methylated BSA (mBSA)/CFA. Seven days later, sorted CD4^+^CCR6^+^ T cells or γδ T cells from the spleens and LNs of these mice were transferred into IL‐23R^−/−^ recipients. After 1.5 h, WT and recipient IL‐23R^−/−^ mice were immunized with mBSA/CFA. AIA was induced with an intra‐articular injection of mBSA seven days later. Mice were sacrificed at day 4 of AIA. Macroscopic scores of both inflamed joints representative of two independent experiments are depicted as mean with SEM for *n* = 3–5 mice per group for each experiment. ***p < 0.001 (*Mann–Whitney test*) compared to both WT and recipients of CD4^+^CCR6^+^ T cells.

Since we detected both IL‐23R(GFP)^+^ γδ T cells and IL‐23R(GFP)^+^CD4^+^CCR6^+^ T cells in the inflamed joints of mice (Fig. 2E and F), we aimed to investigate which of these cells are important for the progression of IL‐23R‐dependent arthritis. Therefore, we adoptively transferred γδ T cells or CD4^+^CCR6^+^ T cells from immunized WT mice into IL‐23R deficient mice and induced AIA in the recipient mice. At day 4 of AIA, the knee joints of the mice were assessed for joint inflammation. Interestingly, while γδ T cells did not affect the disease severity, WT CD4^+^CCR6^+^ T cells were able to induce arthritis in IL‐23R deficient mice (Fig. 7). These data suggest that although both CD4^+^CCR6^+^ T cells and γδ T cells express the IL‐23R and are present at the site of inflammation during the peak of disease, specifically the CD4^+^CCR6^+^ T cells are important for IL‐23R‐mediated joint inflammation.

## Discussion

In this study, we demonstrate that IL‐23R deficient mice are protected from the progression of AIA and have less bone damage. In this context, CD4^+^CCR6^+^ T cells, but not γδ T cells, are crucial for the progressive phase of arthritis. IL‐23R is expressed on γδ T cells and CD4^+^CCR6^+^ T cells, but not on CD8^+^ T cells, during both naïve and arthritic conditions. During the early phases of arthritis, IL‐23R^+^CD4^+^CCR6^+^ T cells were increased in the pLNs of mice and were present in the joints, while IL‐23R^+^ γδ T cells were not present in the joints at this time point. Although IL‐23R^+^CD4^+^CCR6^+^ T cells and CCR7^+^CD4^+^CCR6^+^ T cells were expanded in the lymphoid tissues of IL‐23R deficient mice, IL‐23R^+^CD4^+^CCR6^+^ T cells were still present in the joints of these mice.

IL‐23R^GFP/+^ mice were used for studying the dynamics of IL‐23R^+^ T cells during arthritis. Despite having only one functional IL‐23R allele, these mice had similar susceptibility to arthritis as their WT littermates. This is in line with an earlier study in IL‐23R^GFP/+^ mice, which reported responsiveness of both αβ T cells and γδ T cells to IL‐23 stimulation 26. Furthermore, IL‐23R^GFP/+^ mice had similar susceptibility to EAE as WT mice 11.

In contrast to IL‐23R^GFP/+^ mice, IL‐23R^GFP/GFP^ (IL‐23R^−/−^) mice lack both alleles of the *il23r* and had significantly less severe joint inflammation and damage. This is in line with previous studies in IL‐23p19^−/−^ mice 15, 27 and indicates that IL‐23/IL‐23R signaling is crucial for the progressive phase of AIA. Importantly, both IL‐23p19^−/−^ and IL‐23R^−/−^ mice are also knocked‐out for IL‐39 (IL‐23p19+ Ebi3 heterodimer) pathway 28. Considering the role of this pathway in systemic lupus erythematosus, it is plausible that this pathway could also be involved in the AIA model. Further studies should reveal if this pathway plays a role in AIA and if IL‐39R is expressed on CD4^+^CCR6^+^ T cells.

During the progressive phase of arthritis, the main infiltrating T cells that were found in the joints of WT mice were CD4^+^ and γδ T cells, while considerably lower numbers of CD8^+^ T cells were detected. This suggests that the role of CD8^+^ T cells is limited in this model. Accordingly, a recent study demonstrated that depletion of CD8^+^ T cells in mice did not affect chronic joint inflammation and destruction in the newly generated mouse model antigen‐ and collagen‐induced arthritis 29. Furthermore, IL‐23R was not expressed on CD8^+^ T cells during both naïve and arthritic conditions and CD8^+^ T cell infiltration in the joints was independent of IL‐23R signaling, which further supports the notion that these cells are dispensable for the IL‐23R‐mediated progressive phase of AIA.

Specifically IL‐23R(GFP)^+^CD4^+^CCR6^+^ T cells were significantly increased in the spleen and LNs of IL‐23R^−/−^ mice. One possible explanation could be that these cells accumulate here and could not egress from these tissues to migrate towards the site of inflammation. However, IL‐23R(GFP)^+^CD4^+^CCR6^+^ T cells were still present in the joints of IL‐23R^−/−^ mice and were even slightly higher compared to IL‐23R^GFP/+^ mice. Another possible explanation is that IL‐23R deficient mice have increased thymic output of IL‐23R^+^CD4^+^CCR6^+^ T cells. Supporting this, IL‐23 or RORγt deficiency in mice resulted in defective apoptosis and negative selection of thymocytes during infection 30. It remains to be determined if the increase in IL‐23R^+^CD4^+^CCR6^+^ T cells, due to absence of IL‐23R signaling, is caused by possible changes in the thymic output of these cells.

Although the expression of CCR7 in CD4^+^CCR6^+^ T cells of IL‐23R^−/−^ spleens was similar to WT, their total cell numbers were significantly higher. In addition, treatment of splenocytes with IL‐23 decreased CCR7 gene expression, suggesting a role for IL‐23 in the regulation of CCR7 in CD4^+^CCR6^+^ T cells. It should be noted that we have used whole spleens to investigate the effect of IL‐23 stimulation on CCR7 gene expression and that in addition to CD4^+^ T cells, CCR7 is expressed on other immune cells including CD8^+^ T cells and DCs 31, 32. Since our data demonstrated that CCR7^+^CD4^+^CCR6^+^ T cells, but not CCR7^+^ γδ T cells, were increased in the spleens of IL‐23R^−/−^ mice, it is plausible that IL‐23 regulation of CCR7 expression is cell‐extrinsic, involving other immune cells. Further studies are required to investigate if IL‐23R deficiency specifically leads to an increase in number of CCR7^+^CD4^+^CCR6^+^ T cells alone or (in)directly affects other immune cells as well and what the potential effects are for the function of these cells.

Interestingly, our finding that in contrast to CCR7^+^CD4^+^CCR6^+^ T cells, CCR7^+^ γδ T cells were not increased in the spleens of IL‐23R^−/−^ mice is supported by the study of Vrieling et al. They demonstrated that γδ T cell homing to skin and migration to skin‐draining LNs is not dependent on CCR7 33. This suggests that CCR7 expression is differently regulated in CD4^+^ T cells versus γδ T cells and may have different roles on these cells.

Our study demonstrated that γδ T cells were not important for the progression of arthritis, however their contribution to the generation of pathogenic CD4^+^CCR6^+^ T cells during arthritis cannot be excluded. Indeed, Petermann et al. demonstrated an important role for IL‐23R^+^ γδ T cells in maintaining Th17 cell pathogenicity by antagonizing Treg cell‐mediated suppression of αβ T cells, and the conversion of conventional Th cells into Treg cells 26. Likewise, in experimental autoimmune uveoretinitis, activated γδ T cells could induce IL‐23 production by DCs in vitro and increased the generation of IL‐17A producing αβ T cells 34. These studies suggest a role for γδ T cells in modulation of CD4^+^CCR6^+^ T cell pathogenicity.

We used the entire CD4^+^CCR6^+^ T cell population rather than the IL‐23R^+^ fraction for the adoptive transfer into IL‐23R deficient mice. This was due to technical reasons; since the fraction of IL‐23R^+^ cells within the CD4^+^CCR6^+^ T cell population is relatively small, it was challenging to obtain enough cells for the transfer. However, this raises the question whether all CD4^+^CCR6^+^ T cells are important for joint inflammation or that the IL‐23R^+^ fraction of CD4^+^CCR6^+^ T cells is sufficient. Previously, Ghoreschi et al. demonstrated that adoptively transferred Th17 cells that were generated with IL‐23, induced more severe EAE in RAG^−/−^ recipient mice than Th17 cells that were induced with TGF‐β 35. In addition, Komatsu et al. demonstrated the pathogenic conversion of FOXP3^+^ T cells into IL‐23R expressing Th17 cells that promoted arthritis 36. These studies indicate that IL‐23R expression on CD4^+^ T cells is associated with the pathogenicity of these cells.

The transfer experiments in our study have some technical limitations. Since we have used αCD3 beads for sorting T cells, this may have resulted in activation and increased cytokine production by T cells. Therefore, this does not completely determine the IL‐23R signaling dependency of CD4^+^CCR6^+^ T cells in mediating inflammation in our model. However, the data of the adoptive transfer experiments are in line with Figs. 2E and F, which demonstrate that CD4^+^IL‐23R(GFP)^+^ cells were present in the joints already one day after induction of AIA, in contrast to IL‐23R(GFP)^+^ γδ T cells.

Also, the potential difference in production of IL‐17 between CD4^+^ and γδ T cells, which may have been induced by CD3 activation, could play a role in our transfer experiments, although a previous study demonstrated that lack of IL‐17A had no effect on the severity of AIA 16. Further research is needed to unravel the dependency of the IL‐23R signaling in CCR6^+^CD4^+^ T cells versus γδ T cells using IL‐23R cell type specific knockout mice.

In conclusion, our study demonstrates that IL‐23R^+^ CD4^+^CCR6^+^ T cells are present in the inflamed joints during the early stages of arthritis. Furthermore, CD4^+^CCR6^+^ T cells, but not γδ T cells, are important for IL‐23R‐dependent progression of arthritis. In addition, our study demonstrates a new role for IL‐23R signaling in the regulation of CCR7 expression in the secondary lymphoid tissues.

## Material and methods

### Mice

Eight to twelve weeks old male and female mice were used for the experiments. Knock‐in IL‐23R‐GFP reporter (IL‐23R^GFP/+^) mice were kindly provided by Dr. Mohamed Oukka and Dr. Vijay K. Kuchroo 11. As described previously by Awasthi et al., IL‐23R^GFP/+^ mice were generated by introduction of an IRES‐GFP cassette after exon 8 in the endogenous IL‐23R gene 11. The targeting construct was electroporated into Bruce4 ES cells. Targeted ES cells were injected into BALB/c blastocysts and male chimeras were bred with female C57BL/6 mice. IL‐23R^GFP/+^ mice were bred as homozygotes in our own facility to obtain IL‐23R^GFP/GFP^ (also referred to as IL‐23R deficient or IL‐23R^−/−^ in this paper) and IL‐23R^+/+^ (WT) mice. Food and water were provided *ad libitum*, and mice were kept under specific pathogen‐free conditions. All experiments were approved by the Erasmus MC Dutch Animal Ethics Committee (DEC).

### AIA induction

AIA was induced as previously described 37. Briefly, CFA (H37Ra; BD Difco) was supplemented with heat‐killed mycobacterium tuberculosis (BD Difco) to obtain a final concentration of 5 mg/mL. The supplemented CFA was emulsified with an equal volume of methylated BSA (mBSA). The final concentration of mBSA in the emulsion was 4 mg/mL. Mice were immunized by an intra‐dermal injection of 100 µL of mBSA/CFA emulsion at the tail base. Seven days later, mice received an intra‐articular injection of 6 µL mBSA (60 µg) in saline in both knee joints to induce arthritis. The severity of arthritis in the knee joints was scored macroscopically. After removing the skin from each knee, the joint was scored on a scale from 0–2, where 0 = no inflammation, 1 = mild inflammation, 1.5 = marked inflammation, and 2 = severe inflammation, in increments of 0.25. A score of 0.25 was given when the first signs of swelling and redness were present. The macroscopic scoring of the joint was performed by two blinded observers.

### Histology

Knees were fixed in 10% formalin for 3 days, decalcified in 10% EDTA (pH 7.2) for fourteen days, and subsequently infiltrated and embedded in paraffin 37. Coronal sections of 6 µm thick slices were cut. Paraffin was removed by bringing sections through xylene and gradients of ethanol (100–70%, 5 min per step) and sections were then rinsed in distilled water for 5 min. For assessment of inflammation, sections were stained with hematoxylin for 5 min, washed with tap water for 5–10 min, and stained with eosin for 45 s. Sections were dehydrated in gradients of 70–100% ethanol and xylene (1 min each) and mounted with Entallan (Depex). Images were acquired using NanoZoomer (Hamamatsu Photonics). Histopathological changes in the knee joints were scored in the patella/femur/tibia region on three semi serial sections of the joint, spaced 70 µm apart. Scoring was performed by two blinded observers. Histopathological changes were scored using the following parameters. Infiltration of cells was scored on a scale of 0–3, depending on the amount of inflammatory cells in the synovial cavity (exudate) and synovial tissue (infiltrate). A characteristic parameter in AIA is the loss of bone. This destruction was graded on a scale of 0–3, ranging from no damage to complete loss of the bone structure at four different locations in the patella/femur region resulting in a cumulative score of a maximum of 12.

### Single cell isolation and flow cytometry

Patellae with adjacent synovium was cut into small pieces and incubated for 1–1.5 h at 37°C with 5 µg/mL Liberase^TM^ (collagenase I and II, Sigma‐Aldrich) in Roswell Park Memorial Institute 1640 medium. Single cell suspension of spleens and synovium was prepared using 100 µm cell strainers (Falcon). Erythrocytes in splenic cell suspension were lysed using Gey's solution. Single cell suspension from the LNs was prepared using tubes with 35 µm nylon mesh cell strainer snap cap (Falcon).

Single cell suspensions were incubated in Fc block (anti‐FcγR II and III; clone 2.4G2) for 20 min at 4°C and were subsequently incubated with anti‐mouse CD3, TCR‐γδ, CD45, CCR6 (all from BioLegend), CD4, CD8, and CD62L (all from BD Pharmingen) antibodies for 30 min at 4°C in the dark. For exclusion of dead cells, samples were incubated with Fixable Viability Dye eFluor506 (eBioscience) for 30 min at 4°C in the dark and fixed with 2% paraformaldehyde for 5 min. For intracellular flow cytometry, cells were stimulated using 50 ng/mL PMA, 500 ng/mL ionomycin (Sigma‐Aldrich) and Golgistop (BD Bioscience) as described by the manufacturer for 4 h. Cells were stained with cell surface markers as described above. Fixable Viability Dye eFluor506 (eBioscience) was used to exclude dead cells. After fixation with 2% paraformaldehyde, cells were permeabilized using 0.5% saponin buffer and stained with antibodies against IL‐17A (BD Pharmingen). Cells were acquired on LSRII flow cytometer (BD Biosciences) and analyzed using FlowJo v7.6 software (Tree Star). We have adhered to the guidelines of ‘Guidelines for the use of flow cytometry and cell sorting in immunological studies’ 38.

### Cell sorting and adoptive transfer

WT mice were immunized with mBSA/CFA as described above. Seven days later, single cell suspensions from the spleens and LNs were prepared as described above. For sorting T cells, CD3^+^ cells were pre‐purified with MACS (Miltenyi Biotec) according to manufacturer's instructions. The positively selected cells were used for the FACS. Dead cells were excluded with 4',6‐diamidino‐2‐fenylindool. CD3^+^CD8^−^TCRγδ^−^CD4^+^CD62L^−^CCR6^+^ (referred to as CD4^+^CCR6^+^ T) cells or CD3^+^TCRγδ^+^ (γδ T) cells were sorted. Sorted cells were resuspended in 0.9% NaCl. Per mouse, 1.2×10^5^ CD4^+^CCR6^+^ T cells or 6×10^4^ γδ T cells were injected in the tail vein of age and sex matched recipient mice in a volume of 100 µL. After 1.5 h, recipient mice were immunized with mBSA/CFA, and seven days later, arthritis was induced via an intra‐articular injection of mBSA.

### Cell culture and RT‐PCR

Splenic cell suspensions were prepared as described above. 1×10^5^ cells were cultured in Iscove's modified Dulbecco's medium supplemented with 10% fetal calf serum (Gibco), 2 mM l‐glutamine, 100 U/mL penicillin/streptomycin (Lonza) and 50 µM β‐mercaptoethanol (Sigma‐Aldrich) for 3 days. Cells were cultured in U‐bottom 96‐well plates and left untreated or treated with 50 ng/mL recombinant IL‐23 (R&D Systems). RNA was isolated using the GenElute Mammalian Total RNA Miniprep Kit according to manufacturer's instructions (Sigma Aldrich). RNA was treated with 0.1 U/µL DNAse I Amplification Grade (Invitrogen). cDNA was synthesized using random hexamer primers and 10 U/µL Superscript II (Invitrogen). Primers were designed with ProbeFinder software (Roche Applied Sciences, USA) and probes were used from the Universal Probe Library (Roche Applied Science, USA). 18srRNA (forward primer 5′‐GCAATTATTCCCCATGAACG‐3′; reverse primer 5′‐ GGGACTTAATCAACGCAAGC‐3′; probe 48) was used to normalize gene expression. For CCR7, forward primer 5′‐ CAGGGAAACCCAGGAAAAAC‐3′ and reverse primer 5′‐ATCTTGGCAGAAGCACACCT‐3′ with probe 77 were used. Real‐time PCR was performed using the Viia7 system and data were analyzed using QuantStudio Real‐time PCR software version 1.3 (Applied Biosystems, USA).

### Statistical analysis

Data are expressed as mean ± SEM. Differences between two groups were tested using Mann–Whitney test and differences between multiple groups were tested using Kruskall–Wallis test followed by Dunn's multiple comparison (GraphPad Prism 5). *p*‐values < 0.05 were considered statistically significant.

## Conflict of interest

The authors declare no commercial or financial conflict of interest.

**Abbreviations**
AIA: antigen‐induced arthritis
mBSA: methylated BSA
pLN: popliteal lymph nodes

## Supporting information

Supporting Information
