# Supplementary material for: CD4+CCR6+ T cells, but not γδ T cells, are important for the IL‐23R‐dependent progression of antigen‐induced inflammatory arthritis in mice
Source: Eur J Immunol. 2019 Nov 28;50(2):245–55. doi: 10.1002/eji.201948112 (PMC7028107; doi:10.1002/eji.201948112)
Supplement: Supplementary file 1 — Supporting Information [file EJI-50-245-s001.pdf]

European Journal of Immunology

Supporting Information
for

DOI 10.1002/eji.201948112

Wida Razawy, Patrick S. Asmawidjaja, Anne-Marie Mus, Nazike Salioska, Nadine Davelaar, Nicole Kops, Mohamed Oukka, C. Henrique Alves and Erik Lubberts

CD4^{+}CCR6^{+} T cells, but not $\gamma\delta$ T cells, are important for the IL-23R-dependent progression of antigen-induced inflammatory arthritis in mice

© 2019 The Authors. *European Journal of Immunology* published by WILEY-VCH Verlag GmbH & Co. KGaA, Weinheim.

Supplementary figures

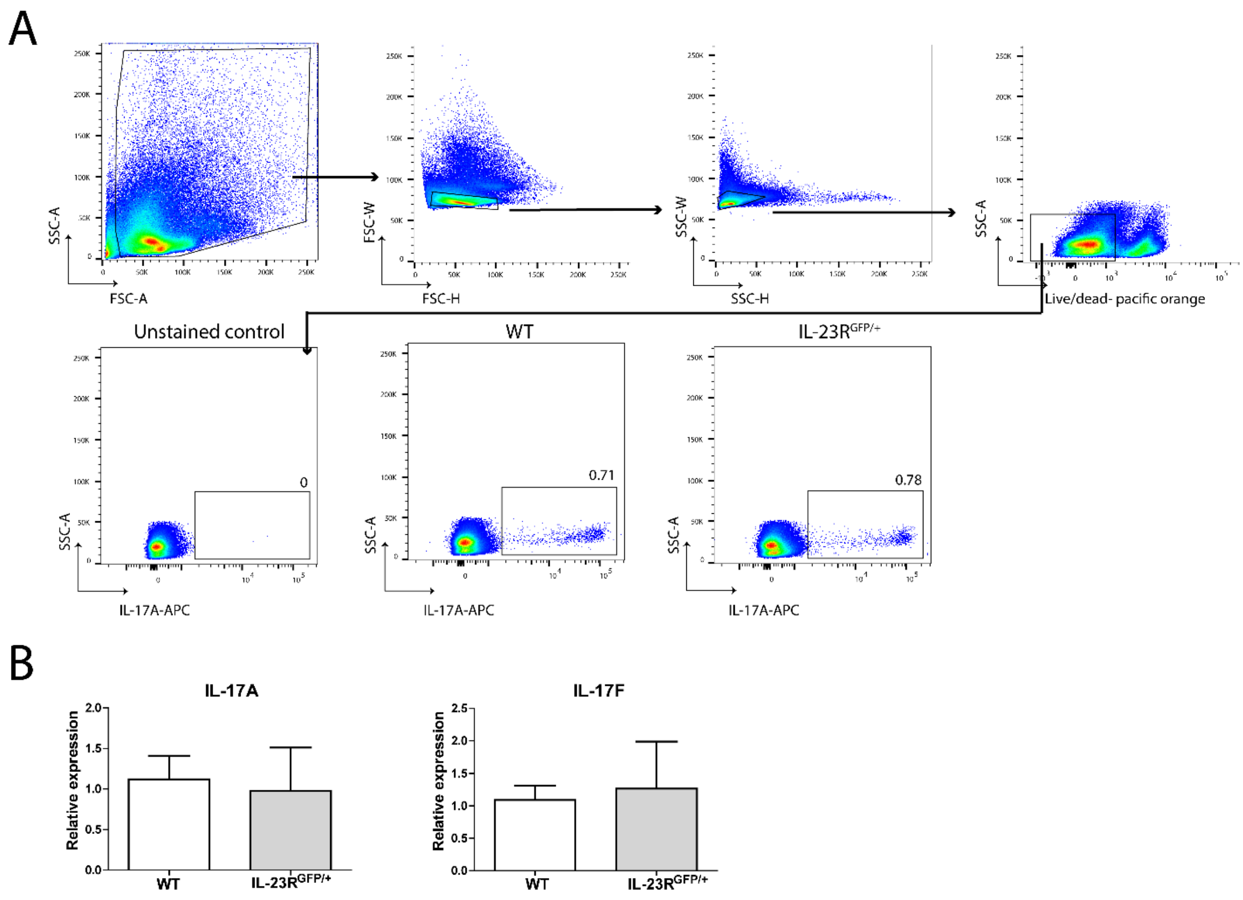

Figure S1. AIA was induced in IL-23R$^{GFP/+}$ and WT mice, and mice were sacrificed at day 4 after arthritis induction. **(A)** Representative FACS plots for IL-17A staining in the spleens of mice at day 4 of AIA after stimulation of cells for 4 hours with PMA/ionomycin. **(B)** IL-17A and IL-17F gene expression in whole spleen assessed by RT-PCR. Representative data of two independent experiments given for n=4 mice per group per experiment. Data are depicted as mean with SEM.

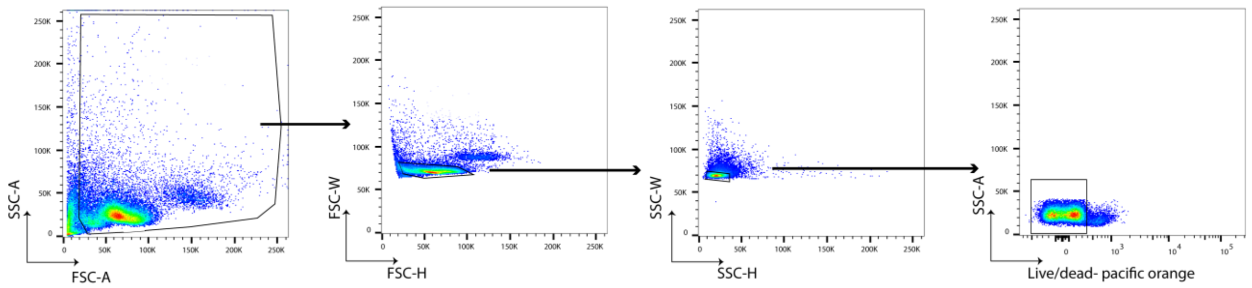

Figure S2. Pre-gating strategy of cells as depicted in figure 2A. AIA was induced in IL-23R$^{GFP/+}$ and WT mice. Naïve and arthritic mice were sacrificed at day 1, 4 or 7 of AIA. IL-23R(GFP) expression was assessed by flow cytometry. WT mice were always used as negative control for GFP signal.

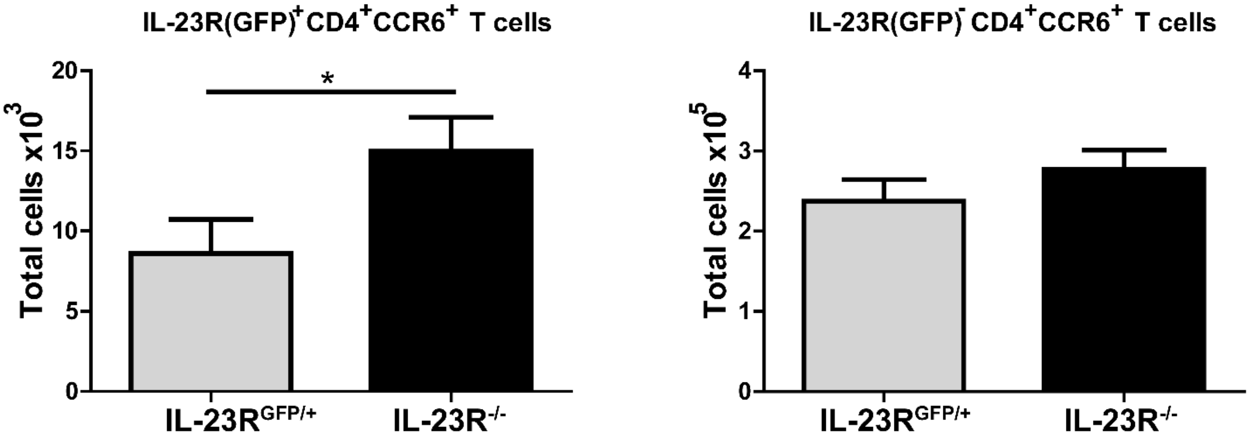

Figure S3. Total cell counts of IL-23R(GFP)$^+$ and IL-23R(GFP)$^-$CD4$^+$CCR6$^+$ T cells in the inguinal LNs at day 4 of arthritis. AIA was induced in WT, IL-23R$^{GFP/+}$ and IL-23R$^{-/-}$ mice and the inguinal LNs were harvested at day 4 after arthritis induction. Total IL-23R$^+$ T cell count was determined using GFP expression by flow cytometry. WT mice were used as negative control for GFP. Representative data of two independent experiments given for n=4 mice per group per experiment. Data are depicted as mean with SEM. *$*p < 0.05$ (Mann-Whitney test)*.

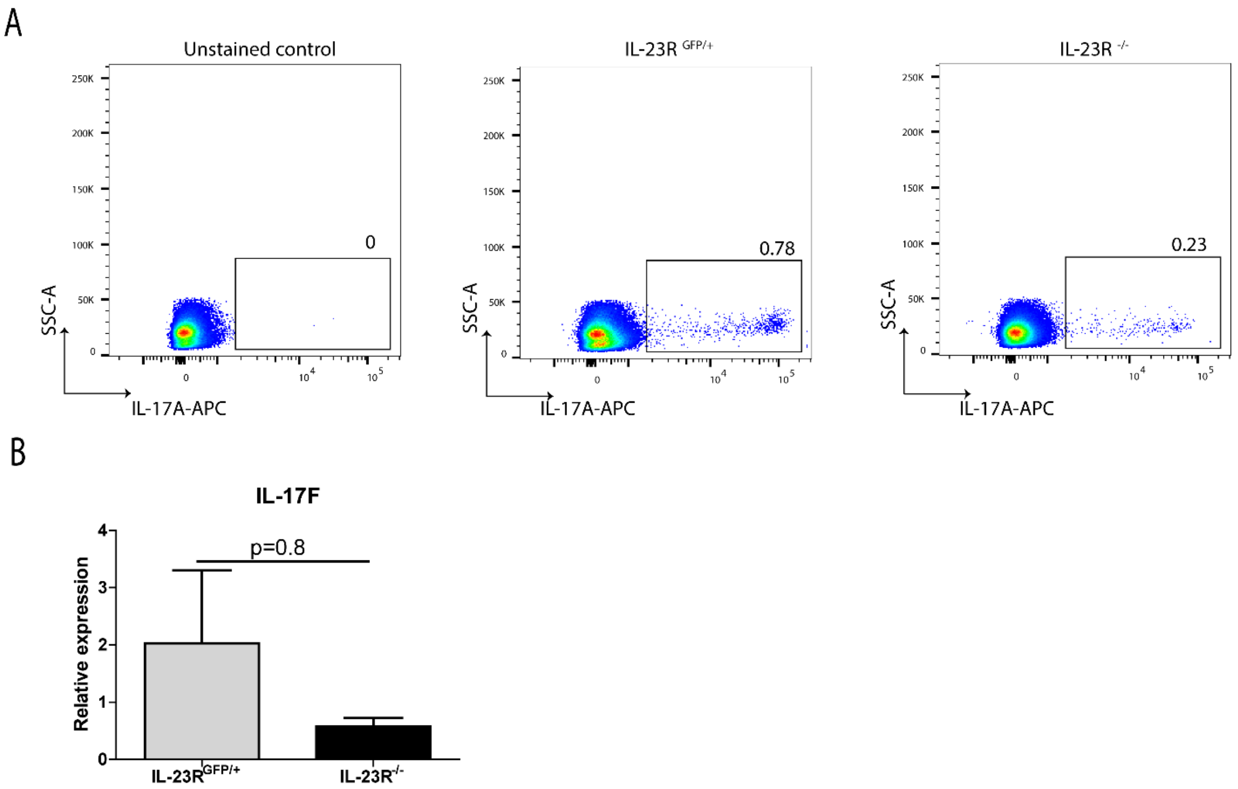

Figure S4. AIA was induced in IL-23R$^{GFP/+}$ and IL-23R$^{-/-}$ mice and the spleens were harvested at day 4 after arthritis induction. **(A)** Representative FACS plots of IL-17A measured in splenocytes after stimulation of cells for 4 hours with PMA/ionomycin. **(B)** IL-17F gene expression in whole spleen assessed by RT-PCR. Representative data of two independent experiments given for n=4 mice per group per experiment. Data are depicted as mean with SEM.

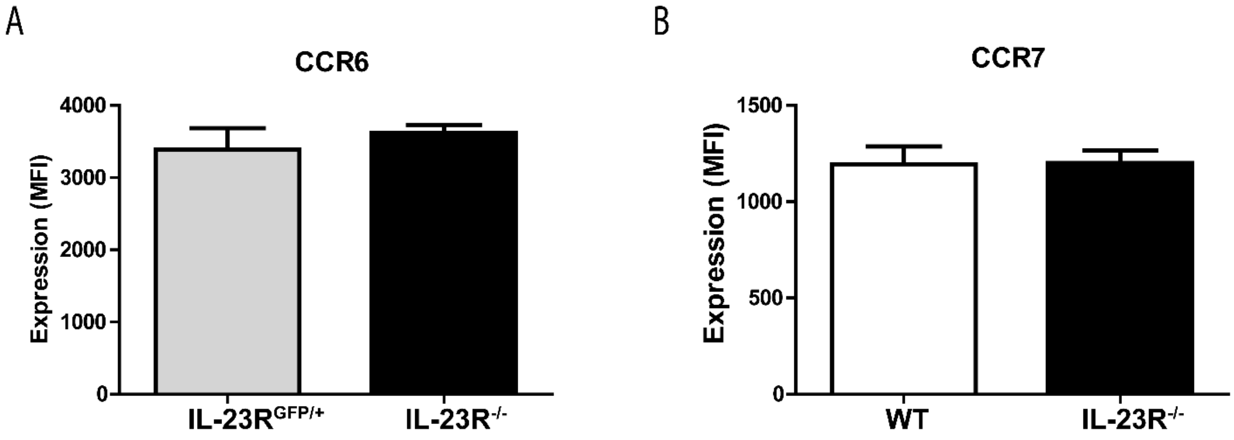

Figure S5. AIA was induced in WT, IL-23R$^{GFP/+}$ and IL-23R$^{-/-}$ mice and the spleens were harvested at day 4 after arthritis induction. **(A)** CCR6 MFI in IL-23R(GFP)$^{+}$CD4^{+}CCR6^{+} T cells. **(B)** CCR7 MFI on total CD4^{+}CCR6^{+} T cells. Data are representative of two independent experiments with n=4 mice per group per experiment. MFI= mean fluorescent intensity.
